# Evaluating Temporal Correlations in Time Series Using Permutation Entropy, Ordinal Probabilities and Machine Learning

**DOI:** 10.3390/e23081025

**Published:** 2021-08-09

**Authors:** Bruno R. R. Boaretto, Roberto C. Budzinski, Kalel L. Rossi, Thiago L. Prado, Sergio R. Lopes, Cristina Masoller

**Affiliations:** 1 Department of Physics, Universidade Federal do Paraná, Curitiba 81531-980, Brazil; brrb12@fisica.ufpr.br (B.R.R.B.); prado@fisica.ufpr.br (T.L.P.); lopes@fisica.ufpr.br (S.R.L.); 2 Department of Mathematics, Western University, London, ON N6A 3K7, Canada; rbudzins@uwo.ca; 3 Brain and Mind Institute, Western University, London, ON N6A 3K7, Canada; 4 Theoretical Physics/Complex Systems, ICBM, Carl von Ossietzky University Oldenburg, 26129 Oldenburg, Germany; kalel.rossi@uni-oldenburg.de; 5 Department of Physics, Universitat Politecnica de Catalunya, 08034 Barcelona, Spain

**Keywords:** ordinal analysis, symbolic analysis, machine learning, time series analysis, permutation entropy, complexity, chaos, noise

## Abstract

Time series analysis comprises a wide repertoire of methods for extracting information from data sets. Despite great advances in time series analysis, identifying and quantifying the strength of nonlinear temporal correlations remain a challenge. We have recently proposed a new method based on training a machine learning algorithm to predict the temporal correlation parameter, α, of flicker noise (FN) time series. The algorithm is trained using as input features the probabilities of ordinal patterns computed from FN time series, $x_{\alpha }^{FN}\left(t\right)$, generated with different values of α. Then, the ordinal probabilities computed from the time series of interest, x(t), are used as input features to the trained algorithm and that returns a value, $\alpha_{e}$, that contains meaningful information about the temporal correlations present in x(t). We have also shown that the difference, Ω, of the permutation entropy (PE) of the time series of interest, x(t), and the PE of a FN time series generated with $\alpha =\alpha_{e}$, $x_{\alpha_{e}}^{FN}\left(t\right)$, allows the identification of the underlying determinism in x(t). Here, we apply our methodology to different datasets and analyze how $\alpha_{e}$ and Ω correlate with well-known quantifiers of chaos and complexity. We also discuss the limitations for identifying determinism in highly chaotic time series and in periodic time series contaminated by noise. The open source algorithm is available on Github.

## 1. Introduction

Thanks to huge advances in data science and computing power, a wide repertoire of time series analysis methods [1,2,3,4,5,6,7] are available for the quantitative characterization of time series and are routinely used in all fields of science and technology, social sciences, economy and finance, etc. Since different methods have different requirements and involve different approximations, no single method can be expected to perform well over all types of data. Therefore, despite huge advances, extracting reliable information from stochastic or high dimensional signals remains a challenge. As any algorithm will return, at least, a number (i.e., a “feature” that encapsulates some property of the time series), in order to interpret the information in the obtained features and to assess the performance of different algorithms, appropriate surrogates [8] or a “reference model” (where the systems that generates the data are known) need to be used. The comparison of the features obtained from the time series of interest with those obtained from surrogate time series or from reference time series allows testing (and even quantifying) some particular property of the time series of interest.

We have recently proposed a new method for estimating the strength of the temporal correlations in a given time series, which uses flicker noise (FN), a fully stochastic process, as the reference model [9]. A FN time series, $x_{\alpha }^{FN}\left(t\right)$, is characterized by a power spectrum $P\left(f\right)\propto 1/f^{\alpha }$, with α being a parameter that quantifies the temporal correlations present in the signal [10]. The method proposed in [9] combines the use of symbolic ordinal analysis [11,12] and machine learning (ML): We utilize the ordinal probabilities computed from FN time series generated with different α values as input features to a ML algorithm. The algorithm is trained to return the value of $\alpha_{e}$, which estimates the real α value of a FN time series from the D! probabilities of the ordinal patterns of length *D*, calculated from the same time series. Then, after the training stage, the ordinal probabilities now computed from a time series of interest, x(t), are provided as input features to the ML algorithm that returns a value, $\alpha_{e}$, which encapsulates reliable information about the strength of the temporal correlations present in x(t). By calculating the difference, Ω, between the permutation entropy (PE) of x(t) and that of a FN time series generated with $\alpha =\alpha_{e}$, $x_{\alpha_{e}}^{FN}\left(t\right)$, we were also able to identify determinism in x(t).

Our approach is, thus, based on reducing a large number of features (with D=6, we have D!=720 ordinal probabilities) to only two: the $\alpha_{e}$ value returned by the ML algorithm; and the permutation entropy, $\bar{S}$, computed with the ordinal probabilities. Dimensionality reduction is a well known technique [13,14,15] that has been used to tackle a variety of problems. With ordinal probabilities, for instance, it is possible to distinguish between noise and chaos by reducing the set of probabilities to only two features—the permutation entropy and the complexity; or the permutation entropy and the Fisher information—as demonstrated in [16,17,18,19]. In our methodology, we not only apply dimensionality reduction but also use a fully stochastic “reference” FN time series: we compare the value of $\bar{S}$ of the time series of interest with that of a FN time series generated with $\alpha =\alpha_{e}$. We have shown that the entropy difference, Ω, may provide good contrast for distinguishing fully stochastic time series from a time series with a degree of determinism [9].

The method we proposed has in fact a large degree of flexibility because, instead of ordinal analysis and the permutation entropy, different symbolization rules [20,21] and different entropies [22,23,24] could be tested. In addition, while we use a simple artificial neural network, other algorithms could be evaluated. Different combinations may provide different results and particular combinations may result in optimized performance for the analysis of particular types of time series.

We have shown that the algorithm returns meaningful information even from time series that are very short: for the synthetic examples considered in [9], we could distinguish whether the dynamics is mainly chaotic or stochastic with only 100 data points. However, an open question is as follows: What are the limitations in terms of the level of chaos, the level of noise and the length of the time series? Here, we address this issue by using as examples the time series generated with the Logistic map, the βx map and the Schuster map. We also address the following questions: Can we distinguish a highly chaotic time series from a stochastic one? Can we identify a periodic signal hidden by noise? In addition, to gain insight into the information encapsulated by $\alpha_{e}$ and Ω, we contrast them with well known quantifiers of chaos and complexity: the maximum Lyapunov exponent and the ordinal-based statistical complexity measure [25].

The organization of the paper is as follows: Section 2 describes the methodology, Section 3 presents the datasets and systems analyzed, Section 4 presents the results and Section 5 presents the discussion and our conclusions.

## 2. Methodology

The methodology proposed in [9] can be described in a few steps:

1. Calculate the ordinal probabilities (OPs) of a large set of FN time series generated with different values of α and use them as features to train a ML algorithm to return the (known) value of $\alpha_{e}$;
2. Calculate the OPs of the time series of interest, x(t), and use them as features to the trained ML algorithm, which returns a value $\alpha_{e}$ (see Section 2.1);
3. Generate a FN time series with $\alpha =\alpha_{e}$ and calculate its permutation entropy (PE), ${\bar{S}}_{\mathrm{FN}}$ (see Section 2.2);
4. Calculate the relative difference, Ω, between the PE of x(t), $\bar{S}$ and ${\bar{S}}_{\mathrm{FN}}$:
  (1) $$\Omega =\frac{|{\bar{S}}_{\mathrm{FN}}-\bar{S}|}{{\bar{S}}_{\mathrm{FN}}};$$
5. Use the value of $\alpha_{e}$ to quantify the strength of the temporal correlations in the time series of interest and use the value of Ω to identify underlying determinism: if Ω≈0, x(t) is mainly stochastic, otherwise there is some determinism.

In the implementation proposed in [9], the probabilities of the 720 ordinal patterns of length D=6 (described in the next section) were used as input features to the ML algorithm. Then, these features were reduced to only two: the scalar value returned by the ML algorithm, $\alpha_{e}$, which quantifies the temporal correlations presented in the time series of interest x(t); and the permutation entropy, $\bar{S}$ (Equation (4)). Then, the value of $\bar{S}$ was compared with the PE of a FN time series generated with $\alpha =\alpha_{e}$ and the relative difference, Ω (Equation (1)), was found to provide contrast for identifying determinism in x(t). The value of Ω can be used to organize a set of time series according to their level of stochasticity (the lower the value of Ω, the larger the stochasticity level) and, by appropriately selecting a threshold value, Ω can be used to classify the time series into two categories: mainly stochastic and mainly deterministic.

### 2.1. Machine Learning Algorithm

A wide range of ML algorithms are available nowadays. Since we want to regress the information of the features (D! probabilities) into one real value, $\alpha_{e}$ (a classical scalar regression problem), an appropriate simple option is a feed forward artificial neural network (ANN). Mathematically, the ANN can de described as follows. Considering a set of inputs X of D! features with output $\alpha_{e}$, the ANN can be sketched by the following:

(2) $$\begin{array}{ccc} X^{\prime } & = & f\;\;(W\;*X\;\;+b\;), \end{array}$$

(3) $$\begin{array}{ccc} \alpha_{e} & = & f^{\prime }\left(W^{\prime }*X^{\prime }+b^{\prime }\right), \end{array}$$

where W and $W^{\prime }$ are matrices of *weights*, b and $b^{\prime }$ are *biases* column vectors, *f* and $f^{\prime }$ correspond to activation functions and the “*” symbols corresponds to a tensor product. In this sense, $X^{\prime }$ is the result of the transformation f(W∗X+b), which can be understood as a new representation of the inputs X. The elements of the tensors $W,\;W^{\prime },\;b,\;b^{\prime }$ are the parameters of the ANN, which are calibrated in the training state. ANNs are well known, and we refer the reader to our previous work [9] for details about the network structure and the training procedure. We remark that our ANN is a fast and automatic tool and it performed well in all the cases we tested, with a computational time and in a standard notebook, of a few seconds for the analysis of time series with $10^{6}$ data points. However, we do not claim that an ANN is an optimal choice, since different ML algorithms may be even more efficient. It is important to notice also that the optimal choice will likely depend on the characteristics of the time series (length, frequency content, level of noise, etc.).

### 2.2. Ordinal Analysis and Permutation Entropy

Ordinal analysis and the permutation entropy were proposed by Bandt and Pompe [11] almost 20 years ago and they are now well-known. For their interdisciplinary applications, we refer the reader to a recent *Focus Issue* [12].

Here, we compute the ordinal patterns of length D=6 with the algorithm proposed in [26]. For D=6, there are 6!=720 possible patterns. The patterns are calculated with an overlap of D−1 data points, i.e., for a time series with *N* data points, N−D ordinal patterns are obtained, which are then used to evaluate the ordinal probabilities P={P(i);i=1,⋯,D!}. Then, the normalized permutation entropy is calculated as follows.

(4) $$\bar{S}=-\frac{\sum_{i=1}^{D!}P\left(i\right)\ln\left[P(i\right)]}{\ln\left[D!\right]}.$$

### 2.3. Quantifiers of Chaos and Complexity

The Lyapunov exponents measure the local divergence of infinitesimally close trajectories, and positive exponents indicate chaos [27]. While there are important challenges when calculating them from high-dimensional and/or noisy data [3,28], in the case of a one-dimensional dynamical system for which its governing equation is known, the Lyapunov exponent, $x_{n+1}=f\left(x_{n}\right)$, can be straightforwardly calculated as follows.

(5) $$\lambda =\lim_{n\to \infty }\frac{1}{n}\sum_{n}\ln\left[|f^{\prime }\left(x_{n}\right)|\right].$$

A popular measure for characterizing complex systems is the statistical complexity measure [25] that takes into account the distance to the most regular state and the most random states of the system. It is defined as follows:

(6) $$C=\bar{S}\left(P\right)Q\left(P,P_{e}\right),$$

where $\bar{S}$ is the entropy and *Q* is the distance between the distribution that describes the state of the system, P, and the equilibrium distribution, $P_{e}$, that maximizes the entropy.

As we use the probabilities of the ordinal patterns [16,29], $\bar{S}$ is the normalized permutation entropy and $P_{e}=\left\{1/D!,1/D!,\cdots ,1/D!\right\}$. The distance between P and $P_{e}$ is calculated with the Jensen–Shannon divergence:

(7) $$Q\left(P,P_{e}\right)=Q_{0}\left\{S\left(\frac{P+P_{e}}{2}\right)-\frac{S(P)}{2}-\frac{S(P_{e})}{2}\right\},$$

where $Q_{0}$ a normalization factor $Q_{0}=-2{\left\{\left(\frac{D!+1}{D!}\right)\ln\left[D!+1\right]-2\ln\left[2D!\right]+\ln\left[D!\right]\right\}}^{-1}$.

## 3. Datasets

**Figure 1:**
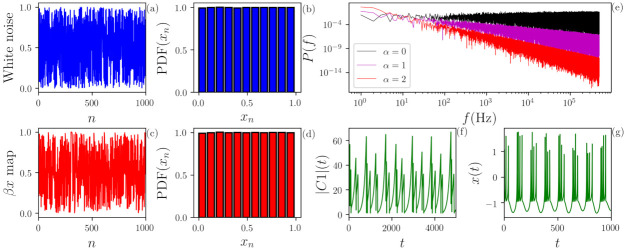
(**a**,**b**) Uniformly distributed white noise and its probability distribution function (PDF). (**c**,**d**) Time series generated by iteration of the βx map with β=2 and its PDF. We observe that the deterministic map depicts a very similar PDF of the white noise. (**e**) Power spectral density (PSD) of flicker noise (FN) α=0 (black), α=1 (magenta) and α=2 (red). By definition, the PSD of the FN decays as $1/f^{\alpha }$. (**f**,**g**) Time evolution of two continuous chaotic systems: (**f**) |C1| of the three-waves system and (**g**) *x* of the Hindmarsh–Rose model.

We analyze time series generated by the following stochastic or deterministic dynamical systems. Typical time series examples are presented in Figure 1.

### 3.1. Flicker Noise

Flicker noise (FN), also called as colored noise, is used for training the ML algorithm. FN time series are stochastic and characterized by a temporal correlation coefficient α. The power spectrum of this signal is given by $1/f^{\alpha }$, and different α values result in different “colors”. We used the open Python library *colorednoise.py* [30,31] to generate FN time series with different α values. An example is shown in panel (e) of Figure 1.

### 3.2. Uniform Noise

Uniform noise is a stochastic process with no memory and uniform distribution in [0,1]. Panels (a) and (b) of Figure 1 depict an example of a uniformly distributed white noise and its PDF, respectively.

### 3.3. Random Walk

A one-dimensional random walk time series is defined by $x_{n+1}=x_{n}+\epsilon_{n}$, where ϵ is a memory-less stochastic Gaussian process with mean 0 and standard deviation 1.

### 3.4. Periodic or Chaotic Signals Contaminated by Noise

In order to generate time series that represent periodic or chaotic signals contaminated by noise, we used the map equation $Z_{n}=\left(1-\eta \right)X_{n}+\eta Y_{n}$. For a periodic signal, $X_{n}$ is the sin map, $X_{n+1}=\sin\left(2\pi n/\tau \right)$ with period τ; for a chaotic signal, $X_{n}$ is the βx map (see below). In both cases, $X_{t}$ is normalized [0,1], $Y_{t}$ is a uniform white noise and η∈[0,1] controls the stochastic component of $Z_{t}$; for η=0, the signal is fully deterministic (periodic or chaotic depending on the map used), while for η=1 the signal is fully stochastic and memory-less.

### 3.5. Logistic Map

The Logistic map is a popular nonlinear dynamical system defined by $x_{n+1}=rx_{n}\left(1-x_{n}\right)$, where *r* is the control parameter that allows us to obtain periodic or chaotic signals.

### 3.6. βx Map

The generalized Bernoulli chaotic map, also known as βx map, is defined by $x_{n+1}=\beta x_{n}\left(\mathrm{mod}1\right)$, where β controls the dynamical characteristic of the map. Panel (c) and (d) of Figure 1 depict the evolution of a βx map with β=2 and its PDF, respectively. Here, we observe that it cannot be visually distinguished from uniform noise; however, the level of chaos can be estimated with the Lyapunov exponent, which has an exact solution in this case: λ=ln(β) [27]. Therefore, the higher the β parameter, the more chaotic the signal is.

### 3.7. Schuster Map

The Schuster map [32] generates intermittent signals with a power spectrum $P\left(f\right)∼1/f^{z}$ and is defined as $x_{n+1}=x_{n}+x_{n}^{z},\;\left(\mathrm{mod}1\right)$, where we used *z* as a parameter control.

### 3.8. Lorenz System

The Lorenz system is also a well-known dynamical system [33], defined by three rate equations: dx/dt=σ(y−x), dy/dt=x(R−z)−y and dz/dt=xy−bz. Here we use typical parameters σ=16, R=45.92 and b=4 that generate chaotic trajectories.

### 3.9. Rossler System

The Rossler system [34] is also a well-known dynamical system, defined by the equations dx/dt=−y−z, dy/dt=x+ay and dz/dt=b+z(x−c). Here, we used typical parameters for generating chaotic trajectories: a=b=0.2 and c=5.7.

### 3.10. Three Waves System

This system is composed of three first-order autonomous differential equations written in terms of the complex slowly varying wave amplitude [35] $dC_{1}/dt=\gamma_{1}C_{1}+C_{2}C_{3}$, $dC_{2}/dt=\gamma_{2}C_{2}-C_{1}C_{3}^{*}+i\delta C_{2}$ and $dC_{3}/dt=\gamma_{3}C_{3}-C_{1}C_{2}^{*}$, where $C_{j}\in C$ and we used $\gamma_{1}=1$, $\gamma_{2}=\gamma_{3}=-17$ and δ=2. The chaotic dynamics can be observed at the amplitude variations of $|C_{1}|$, which is depicted in panel (f) of Figure 1.

### 3.11. Hindmarsh–Rose Model

The Hindmarsh–Rose model [36] is a set of three rate equations that models the neural activity. An example is shown in panel (g) of Figure 1. The equations are $dx/dt=y-ax^{3}+bx^{2}-z+I$, $dy/dt=c-dx^{2}-y$ and $dz/dt=r[s\left(x-x_{r}\right)-z]$ and the parameters used are a=c=1, b=3, s=4, $x_{r}=-8/5$ and r=0.006.

## 4. Results

**Figure 2:**
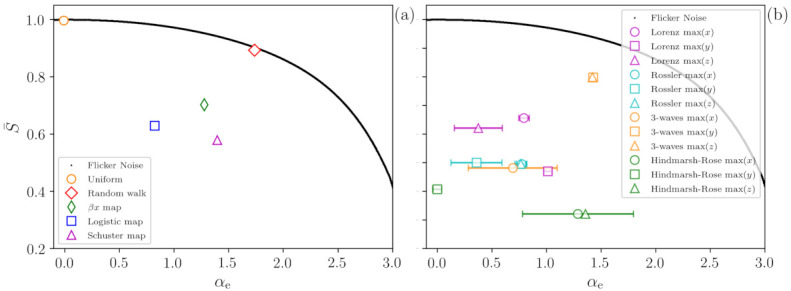
Characterization of stochastic and chaotic time series in the plane (normalized permutation entropy $\bar{S}$; correlation coefficient α returned by the ML algorithm). Panel (**a**) presents results for discrete systems. The black line represents a set of 10,000 FN signals and $\bar{S}$ decreases as the temporal correlation increases. The stochastic cases (uniform noise and random walk) are very close to the FN signals. Otherwise, the chaotic signal (βx, logistic and Schuster maps signals) depict a lower $\bar{S}$ compared to the FN signals. Panel (**b**) presents results for continuous systems. Here, we analyze the sequence of maxima of each variable and, again, we observe that the vertical distance to the FN curve reveals that the time series are not fully stochastic. All time series posses $N=2^{14}$ points and the error bars represent the standard deviation over 1000 time series generated with different initial conditions or noise seeds. Small error bars are not shown.

A demonstration of the methodology is presented in Figure 2 that shows, in the plane ($\bar{S}$, $\alpha_{e}$), the values obtained from time series generated with the dynamical systems described in the previous section. All the time series analyzed possesses $N=2^{14}$ points and the error bars represent the standard deviation over 1000 time series generated with different initial conditions or noise seeds.

Figure 2a presents results for discrete systems, while Figure 2b, for continuous systems. In both panels the black line represents FN signals generated with different values of α, which are perfectly recovered by the ANN (that returns a value $\alpha_{e}$ equal to α). As expected, for $\alpha_{e}=0$, $\bar{S}\approx 1$ since the FN signal is white noise [37]. For $\alpha_{e}>0$, some ordinal patterns occur in the time series more frequently than others, and the value of $\bar{S}$ decreases.

In panel (a), the orange circle represents time series of uniform white noise, and the ANN returns the correct value $\alpha_{e}=0$ (There is almost no dispersion in the returned value of $\alpha_{e}=0$, therefore, the error bar is not shown). The red diamond represents random walk signals; for them, the ANN returns $\alpha_{e}\approx 1.75$ (Again, there is almost no dispersion in the value of $\alpha_{e}$). We observe that the red diamond is very close to the black line, providing a clear indication of the highly stochastic nature of a time series generated by a one-dimensional random walk.

On the other hand, when we analyze chaotic signals (time series from βx map with β=2, from logistic map with r=4 and from Schuster map with z=0.5), we observe that the distance between the symbols and the FN noise curve (black line) allows identifying the signals as not fully stochastic, i.e., the distance to the FN curve uncovers determinism in the signals.

For continuous dynamical systems, the results obtained with ordinal analysis strongly depend on the lag time between the data points, which can be performed in multiple ways, for instance using the maxima of each variable or even the first minimum of the mutual information [38]. While this dependence with the lag allows identifying different time scales in a complex signal [39,40], it renders it difficult to compare different signals. Therefore, for the continuous dynamical systems considered (Lorenz, Rossler, 3-waves and Hindmarsh–Rose), the time series analyzed here are the sequence of maxima of each variable of the system. Due to the fact that the variables obey different equations and have oscillations with different properties, we can expect obtaining different values of $\alpha_{e}$. Despite a large dispersion in the values obtained, it can be observed in Figure 2b that all the systems depict a substantial distance to the FN curve, clearly revealing that they are not fully stochastic. The dispersion in the permutation entropy values is of the order of 1%; therefore, vertical error bars are not shown.

### 4.1. Comparison with Standard Quantifiers

Next, we compare, for the three chaotic maps, the quantifiers obtained with our approach, $\alpha_{e}$ and Ω, with well-known quantifiers of chaos and complexity including the Lyapunov exponent, λ and the ordinal statistical complexity, *C* (described in Section 2.3).

**Figure 3:**
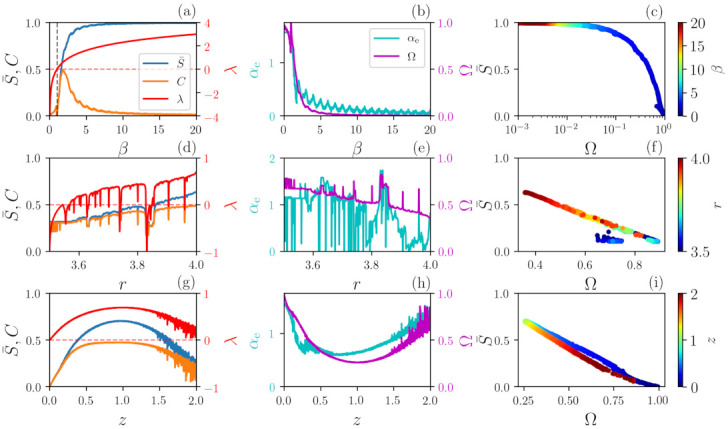
Analysis of deterministic signals generated by chaotic maps. First column depicts the normalized permutation entropy $\bar{S}$, the complexity *C* (left vertical scale) and the Lyapunov exponent λ (right scale). Second column depicts the new quantifiers $\alpha_{e}$ (left scale) and Ω (right scale). The third column presents $\bar{S}$ vs. Ω; the color codes represent the control parameter of each map. Panels (**a**–**c**) illustrate the case of βx map as a function of β; (**d**–**f**) show the Logistic map as a function of *r*; and (**g**–**i**) depict the Schuster map as a function of *z*.

The results are depicted in Figure 3. Panels (a), (b) and (c) show results for the βx map. For β<1, the map is not chaotic since λ=ln(β)<0. In this range, Ω decreases while $\alpha_{e}$ remains constant. The βx map is chaotic for β>1, since λ>0. At β=1, Ω and $\alpha_{e}$ vary abruptly and both decreases as β increases. There is negative correlation between $\bar{S}$ and Ω and also between λ and Ω. If β is too large, Ω≈0 and $\bar{S}\approx 1$ determinism can no longer be identified. We note that the small oscillations of $\alpha_{e}$ capture the changes in dynamics when β approaches an integer number since the PDF of βx is homogeneous for integer β but becomes inhomogeneous for non-integer values [41], which is not clearly observed in the other quantifiers.

In panels (d), (e) and (f) for the logistic map, periodic windows embedded in chaotic regions are detected in the Lyapunov exponent, and the quantifiers $\bar{S}$, *C* and $\alpha_{e}$ also show abrupt variations. We note that Ω identifies the deterministic nature of the signals since, for the entire interval of *r*, Ω remains well above zero and $\bar{S}$ remains well below one.

Similar results are found for the Schuster map, panels (g), (h) and (i): Ω is anti-correlated with λ and confirms that the signal is always deterministic since relatively large Ω values are observed for the entire interval of *z*. Even though a signal generated by the Schuster map has the same power spectrum as Flicker noise, its $\alpha_{e}$ value varies non-monotonically with *z* [32], contrary to the line $\alpha =\alpha_{e}$ that is obtained from FN signals. This is due to the fact that signals generated by the Schuster map and FN signals have different sets of ordinal probabilities.

### 4.2. Influence of the Length of the Time Series

**Figure 4:**
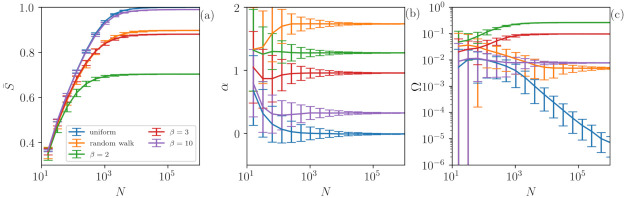
The role of time series length *N* is studied using $\bar{S}$ (**a**), the output of the ANN $\alpha_{e}$ (**b**) and Ω (**c**) for both chaotic and stochastic signals. The results are stable for $N\geq 10^{4}$, indicating the robustness of our methodology. Here, we analyze βx time series and also uniform noise and random walk signals. Despite having similar values of $\bar{S}$, our method is able to distinguish between the chaotic and stochastic cases even for N≤D!=720 (number of ordinal patterns) for β=2 and β=3. However, for β=10 the chaoticity is too high (λ=ln10) therefore existing a great local divergence of the trajectory. In this case, the deterministic nature cannot be detected since the ordinal probability distribution is as uniform as a distribution of a white noise signal with $\bar{S}\approx 1$.

The results so far indicate that our methodology is very precise for characterizing chaotic and stochastic signals when long times series are analyzed (in Figure 2 and Figure 3 $N=2^{14}$). However, an important question is the following: What is the role of the time series size in the analysis? In order to address this issue, we investigate both stochastic and chaotic time series with different lengths *N*. Figure 4 displays the role of time series length *N* in $\bar{S}$ (a), in the output of the ANN $\alpha_{e}$ (b) and in Ω (c) for chaotic and for stochastic signals. Here, chaotic time series are represented by signals generated with the βx map, while stochastic ones are given by uniform white noise and a random walk.

One can observe that, in general, for a small number of data points (N<100), our method is unable to distinguish the signals as all of them are overlapping. Interestingly, for N≈100, our method is able to distinguish the chaotic signal generated by the β map with β=2 (green lines), since Ω depicts values that are higher than those of the other signals. As *N* is increased, the signal of β=3 is also separated and characterized as chaotic. Surprisingly, for N<D!=720 (the number of features), we are already able to distinguish the chaotic signals of β=2 and β=3 from the stochastic ones (uniform and random walk) by using the quantifier Ω. The results improve as the number of points increases and, for $N\geq 10^{4}$, the analysis stabilizes. Here, an important point is that even if the chaotic and stochastic signals have similar permutation entropy values ($\bar{S}$), Ω is able to capture their difference. On the other hand, if the chaoticity of the signal is too high, as observed for β=10 (purple lines), $\bar{S}\approx 1$. In this case the ordinal probabilities of the signal are as uniform as those of white noise, which means that we are unable to identify determinism.

**Figure 5:**
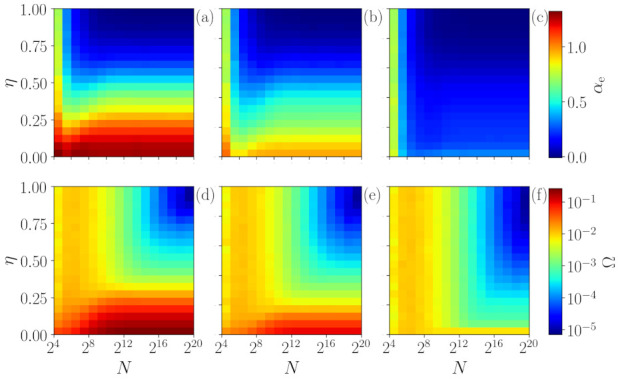
Robustness of the method to the application of additive noise in the βx map. The upper row (**a**–**c**) depicts the $\alpha_{e}$ values in color-code as a function of the percentage of noise η and the series’ size *N* for β=2,3,10, respectively. The lower row (**d**–**f**) shows Ω for the same simulations.

Figure 5 shows that the above observations remain robust when noise is added to the chaotic signal. As explained in Section 3.4, we control the amount of noise in the analyzed time series by varying the parameter η. If the time series is too long and has too much noise, Ω does not identify determinism (blue region). On the other hand, if the time series is not long, Ω identifies determinism even when there is no determinism for η=1 (orange region). Therefore, for a correct interpretation of the information contained in the value of Ω, we need to compare it with the value of $\bar{S}$ obtained from “reference” time series of the same length as the time series of interest, generated by a known stochastic process such as Flicker noise.

### 4.3. Analysis of Periodic Signals Contaminated by Noise

**Figure 6:**
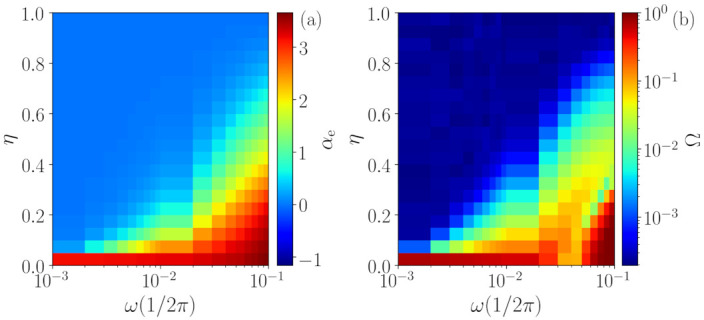
Analysis of periodic signals contaminated with white noise using the temporal correlation $\alpha_{e}$ (**a**) and the quantifier Ω (**b**). The results show that, when the noise strength, η, increases, the deterministic nature of the periodic signal gradually vanishes and, for large enough η, only stochastic dynamics is identified. However, the frequency ω=2π/τ of the signal is important, because low frequency signals are identified as stochastic at lower noise levels. Moreover, even when the signal is characterized as stochastic, a nonzero temporal correlation can be estimated.

Another challenge to our methodology is the identification of periodic signals with added noise. The results obtained when varying the parameter η (see Section 3.4) are depicted in Figure 6, where panel (a) shows the temporal correlation $\alpha_{e}$ returned by the ANN and panel (b) shows the quantifier Ω, both as a function of η. Here, the periodic signals with $N=2^{14}$ points and different frequencies ω are analyzed.

First, one can observe that, for η=0.0, all signals are characterized as deterministic (high value of Ω) with a high temporal correlation (high value of $\alpha_{e}$), which is expected from a periodic signal. Secondly, as we can also expect for η=1.0, all signals are identified as stochastic and memory-less (zero temporal correlation), since the added noise is white. However, for intermediate values of η, an interesting behavior is observed. The frequency of the signal is indeed important, because periodic signals with low frequencies are characterized as stochastic time series for lower values of η (panel (b)). For instance, for $\omega <10^{-2}$, the signal is characterized as noisy even for very small values of η, while for signals with $\omega \approx 10^{-1}$, the deterministic nature of the original time series is kept for η<0.35. As expected, the temporal correlation of the signals $\alpha_{e}$ (panel (a)) also depends on the frequency ω=2π/τ. As η is increased, the higher the frequency of the signal, the slower the way $\alpha_{e}\to 0$ (i.e., high-frequency signals loss memory slower than low-frequency signals). Since the noise being added is white and thus, is memory-less, one can expect that $\alpha_{e}\to 0$ and Ω→0 for η→1. However, we see that the temporal correlation $\alpha_{e}$ can be high even when Ω is very small. Therefore, these signals are first identified as noisy with non-zero time correlation, and, subsequently, they are identified as noisy with no time correlation.

**Figure 7:**
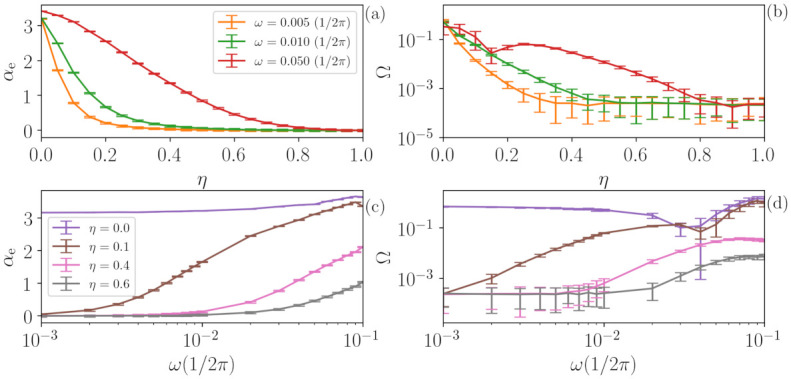
The effects of noise on a periodic signal do not depend on the noise’s specific realization. The error bars indicate the dispersion over 1000 analyses with different realization of the added noise. Here, the upper row represents three examples of $\alpha_{e}$ (**a**) and Ω (**b**) as a function of η for a fixed value of ω. The lower row (**c**,**d**) shows three examples as a function of ω for fixed values of η following the same representation. The dispersion in all cases is sufficiently low that the trends discussed in Figure 6 remain.

These results and conclusions are robust for different realizations of the noise, as is shown in Figure 7, which depicts curves with fixed values of ω (upper row) as a function of η. Panel (a) shows the temporal correlation coefficient $\alpha_{e}$, while panel (b) shows the quantifier Ω. Following the same idea, the lower row shows three examples with fixed η values as a function of the periodic signal frequency ω. In all cases, the error bars represent the dispersion over 1000 different noise realizations, and we can observe that the trends discussed previously persist over different noise realizations.

### 4.4. Analysis of Two Stochastic Processes

**Figure 8:**
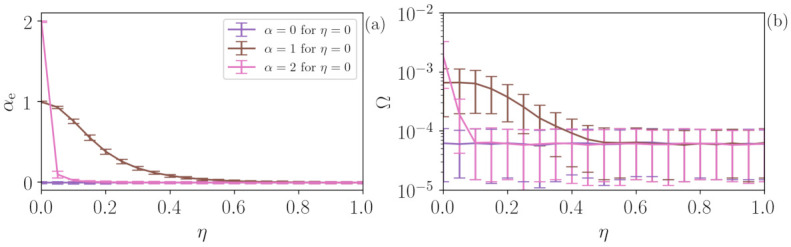
Analysis of FN time series contaminated with uniform noise. (**a**) The addition of uniform noise decreases the $\alpha_{e}$ predicted by the ML algorithm, leading from the value α of the FN time series to value to $\alpha_{e}=0$ for uniform noise. η also decreases the value of Ω (**b**), revealing the increase in the degree of stochasticity of the time series. The addition of uniform noise is, thus, similar in FN and in deterministic time series.

In experimental stochastic systems, two (or more) stochastic dynamics are often present. In order to test the performance of the algorithm in this situation, we consider the same approach as in the previous section, $Z_{n}=\left(1-\eta \right)X_{n}+\eta Y_{n}$, but now $X_{n}$ is not a periodic signal but a Flicker noise. The results are depicted in Figure 8, where panels (a) and (b) present $\alpha_{e}$ and Ω, respectively, and are calculated from 1000 FN time series with α=0 (purple), α=1 (brown) and α=2 (pink). For η=0 (FN noise) $\alpha_{e}=\alpha$, as η increases due to the influence of the uniform white noise, $\alpha_{e}$ decreases to 0 and α=2 (pink line) decays faster due to the slower dynamics, while α=0 (purple) does not change since there is no time correlation in both $X_{n}$ and $Y_{n}$. Ω is extremely low for all cases (<0.01) identifying the full stochasticity of the time series.

## 5. Discussions and Conclusions

We have analyzed stochastic and deterministic time series using an algorithm [9] that automatically reduces the dimensionality of the feature space from 720 probabilities of ordinal patterns to 2 features: the degree of stochasticity (Ω) and the strength of the temporal correlations ($\alpha_{e}$). We have analyzed the performance and limitations of the algorithm, presenting applications to different datasets, including highly chaotic and periodic signals with added white noise.

For the analysis of chaotic time series, we have shown that $\alpha_{e}$ and Ω are able to capture the rich dynamics that chaotic systems can depict, where the transitions between periodic windows and chaos are evident. In general, negative correlation between the Lyapunov exponent and Ω and between the permutation entropy and Ω were found. For highly chaotic signals, when the time evolution of the system is very similar to a stochastic process, $\bar{S}\approx 1$ and Ω≈0 and our methodology characterizes highly chaotic signals as stochastic ones.

In addition, we have studied periodic signals contaminated with noise. In this case, our method captures the transition from deterministic time series to stochastic ones. However, we have shown that the period of the signal is indeed important, with fast signals being identified as deterministic even with large noise. We have found that when the noise contamination increases, periodic signals lose their deterministic feature but preserve a nonzero temporal correlation.

For future work, it will be interesting to analyze whether the performance of our methodology can be improved by using different lengths of the ordinal pattern, *D*, or different lags between the data points that define the ordinal patterns. It is well known that the ordinal patterns distribution varies with the time scale of the analysis [19] and it will also be interesting, for future work, to address the relevant question of whether our method is able to estimate, from the same time series, different values of $\alpha_{e}$ by using different lags.

We remark that the automatic and easy-to-use time series analysis tool that we propose is here is freely available at [42]. We believe that it will be a valuable contribution to the wide repertoire of time series analysis tools that are available nowadays. Many applications are foreseen. As an example, for ultra-fast optical random number generation [43,44,45], it is crucial to generate optical chaotic signals that are as uncorrelated and as ‘‘pseudorandom’’ as possible, for which its deterministic nature is hidden by noise-like properties. Many different setups have been proposed to generate such broad-band, high-entropy optical signals [46,47,48,49,50]. Our algorithm allows an automatic comparison of the strength of the correlations and the level of randomness of signals generated by different setups. Moreover, the algorithm may be used to identify, in a given experimental setup, the optimal operation conditions and parameters that produce optical signals with the lowest temporal correlations (lowest $\alpha_{e}$) or with highest level of randomness (lowest Ω).

## Data Availability

All codes and datasets relevant to our methodology are freely available at [42].
